# On the Question of Operative Interference in the Case of Fibroid Tumours of the Uterus

**Published:** 1898-07-02

**Authors:** James Oliver

**Affiliations:** Physician to the Hospital for Women, London


### Medical Progress and Hospital Clinics.

[*The Editor will be glad to receive offers of co-operation and contributions from members of the profession. All letters should be addressed to* The Editor, at the Office, 28 & 29, Southampton Street, Strand, London, W.C.]

### ON THE QUESTION OF OPERATIVE INTERFERENCE IN THE CASE OF FIBROID TUMOURS OF THE UTERUS.

By James Oliver, M.D., F.R.S. (Edin.), F.L.S., Physician to the Hospital for Women, London.

With very little immediate risk to the life of the patient, the abdominal cavity may in these days be opened; but the operator who recommends and undertakes to perform this operation for the removal of some insignificant and unoffending innocent growth should not banish from his consideration the possible after-effects of such a procedure, since these, even when the convalescence after operation has progressed most satisfactorily, may in later days prove a source of trouble to, and endanger the life of, the individual. In consequence of a simple exploratory incision of the abdominal walls, the intestines may become adherent to the wound, and the risk of intestinal agglutination becomes more and more augmented the more extensively the peritoneum has been lacerated and ligatured. The ligatures left in the abdominal and pelvic cavities do not always remain inert; occasionally they make their way into the bowel and bladder, and in the latter organ they may form the nucleus of a stone. The patient, therefore, who submits to hysterectomy or myomectomy for fibroid tumour of the uterus runs the risk immediately of losing her life, and should the operation be successful, the risk remotely of becoming the subject of a disorder which may prove more troublesome than that for which she seeks relief. Having watched for many years the growth and behaviour of tumours of this class in a very large number of individuals, I am confident that it is unnecessary and unjustifiable to recommend operative interference indiscriminately for their removal. The medical adviser of a patient who suffers from fibroid tumour of the uterus should conscientiously weigh all the facts for and against operation before advising such, and in thus deliberating it behoves him to remember the high trust which is placed in him. If, after due consideration, it be deemed expedient that the growth should be removed, it is incumbent upon the operator to perform that operation which will expose the patient to the least amount of risk, and is likely to prove most salutary. Supra-vaginal hysterectomy, when feasible, is a more commendable operation than pan-hysterectomy, and of the former operation the method whereby the stump is dealt with extra-abdominally is, from the patient's point of view, much more laudable than that in which the intra-peritoneal method of treating the stump is adopted. When the extra-abdominal method of treating the stump is adopted, it is almost invariably possible to complete the operation without leaving any ligatures in the abdomino-pelvic cavity, and without producing any breaches in the peritoneum other than those which are included in the abdominal wound, and so far as the patient is concerned it is impossible to over-estimate the value of these attainments.

